# Accelerated instability testing reveals quantitative mass spectrometry overcomes specimen storage limitations associated with PD-L1 immunohistochemistry

**DOI:** 10.1038/s41374-019-0366-y

**Published:** 2020-01-02

**Authors:** Alexander Haragan, Daniel C. Liebler, Dimple M. Das, Michael D. Soper, Ryan D. Morrison, Robbert J. C. Slebos, Bradley L. Ackermann, Jeff A. Fill, Andrew E. Schade, John R. Gosney, Aaron M. Gruver

**Affiliations:** 1 Institute of Translational Medicine, University of Liverpool, Liverpool, UK; 2 Protypia, LLC, Nashville, TN USA; 3 Lilly Research Laboratories, Eli Lilly and Company, Indianapolis, IN USA; 4 H. Lee Moffitt Cancer Center, Tampa, FL USA

**Keywords:** Predictive markers, Predictive markers

## Abstract

Immunohistochemistry (IHC) using formalin-fixed, paraffin embedded (FFPE) tissue is limited by epitope masking, posttranslational modification and immunoreactivity loss that occurs in stored tissue by poorly characterized mechanisms. Conformational epitopes recognized by many programmed-death-ligand-1 (PD-L1) IHC assays are particularly susceptible to degradation and provide an ideal model for understanding signal loss in stored FFPE tissue. Here we assessed 1206 tissue sections to evaluate environmental factors impacting immunoreactivity loss. PD-L1 IHC using four antibodies (22C3, 28-8, E1L3N, and SP142), raised against intracellular and extracellular epitopes, was assessed in stored FFPE tissue alongside quantitative mass spectrometry (MS). Global proteome analyses were used to assess proteome-wide oxidation across an inventory of 3041 protein groups (24,737 distinct peptides). PD-L1 quantitation correlated well with IHC expression on unaged sections (*R*^2^ = 0.744; *P* < 0.001), with MS demonstrating no loss of PD-L1 protein, even in sections with significant signal loss by IHC impacting diagnostic category. Clones 22C3 and 28-8 were most susceptible to signal loss, with E1L3N demonstrating the most robust signal (56%, 58%, and 33% reduction respectively; *p* < 0.05). Increased humidity and temperature resulted in significant acceleration of immunoreactivity loss, which was mitigated by storage with desiccant. MS demonstrated only modest oxidation of 274 methionine-containing peptides and aligned with IHC results suggesting peptide oxidation is not a major factor. These data imply immunoreactivity loss driven by humidity and temperature results in structural distortion of epitopes rendering them unsuitable for antibody binding following epitope retrieval. Limitations of IHC biomarker analysis from stored tissue sections may be mitigated by cost-effective use of desiccant when appropriate. In some scenarios, complementary MS is a preferred approach for retrospective analyses of archival FFPE tissue collections.

## Abstract (web summary)

PD-L1 IHC was evaluated in stored tissue with mass spectrometry (MS). Increased humidity and temperature resulted in considerable immunoreactivity loss, particularly among antibodies recognizing extracellular and discontinuous epitopes, which could be partially mitigated with the use of desiccant. However, even in significantly affected tissues MS was able to quantitate PD-L1 expression and assess peptide oxidation. These results suggest MS is suited for biomarker assessments using stored sections—a sample type commonly encountered in oncology research.

## Introduction

Formalin-fixation followed by paraffin embedding is the most widely used method for preparing and preserving tissue specimens for clinical and research purposes world-wide. Obtaining, handling, and storing formalin-fixed, paraffin embedded (FFPE) tissue sections presents unique challenges to global pharmaceutical research and development. Standard tissue collections, often influenced by geography and local regulations, can be limiting to the extent that only sectioned tissues adhered to glass slides are available for analysis. This impacts robust biomarker development, since stored FFPE tissue is susceptible to degradation that alters both protein and nucleic-acid assays [1–5]. Recent approved biologics targeting the PD-1 (programmed cell death protein 1) and programmed-death-ligand-1 (PD-L1) immune checkpoint axis utilize IHC for PD-L1 expression as a complementary or companion diagnostic [6, 7], and PD-L1 immunoreactivity on tumor cells (TC) has been shown to decrease during tissue storage [1, 8, 9]. The quantity of PD-L1 expression is crucial for the prescribing of the associated immuno-modulatory drugs; therefore, the instructions for use of the FDA approved PD-L1 IHC assays recommend testing tissues as soon as possible, but no later than 1–6 months for most applications [10–13].

Previous studies have found antigen degradation to be both antigen specific and condition dependent; therefore, a detailed understanding of how specific handling and storage conditions affect novel assays warrants greater appreciation in earlier stages of development. FFPE tissue sections are more prone to loss than tissue blocks [14, 15] and differing environmental conditions can accelerate or mitigate loss of antigenicity [3, 16–21]. However, there appears to be no single factor responsible for antigen degradation: fixation methodology, storage time, ultraviolet A exposure, oxidation, humidity, and temperature are all implicated [16, 22–24]. The difficulty of studying antigen degradation is further compounded by the lack of defined metrics for predicting and measuring it with respect to specific assays.

An alternative approach to IHC for protein quantitation in archival tissues is analysis by targeted mass spectrometry (MS) [25–27]. This method measures protein-specific peptide sequences extracted from unstained FFPE sections and enables precise quantitative comparisons of protein abundances within individual samples and across cohorts. The application of targeted MS to measure PD-L1 and several other immune checkpoint and immunoregulatory proteins in archival FFPE sections from melanomas has been described [27]. In this context, MS and IHC measurements of PD-L1 were largely concordant, except for samples in which PD-L1 was found to be highly glycosylated by MS.

Here we describe a reproducible model of accelerated antigen instability testing and use it to address questions of how immunoreactivity is affected in stored tissue. We specifically evaluate the role of protein oxidation in antigen degradation, how different model IHC assays respond to accelerated conditions, and how MS may be used to evaluate and overcome the limitations of IHC in detecting PD-L1 in stored FFPE tissue sections in the research setting.

## Materials and methods

### Specimens studied

FFPE tissue blocks of non-small cell lung carcinoma (NSCLC), gastric carcinoma, placenta, and tonsil tissue were commercially acquired from Asterand Bioscience (Detroit, MI, USA), US Biomax (Rockville, MD, USA), Tristar Technology Group (Washington, DC, USA), and Indiana University Health Methodist Hospital biobank (Indianapolis, IN, USA) in either tissue microarray (TMA) or whole section format. Tissue blocks were obtained between 2012 and 2018. Some 1206 tissue sections were evaluated from 35 gastric carcinomas [tubular adenocarcinoma], 10 NSCLCs [2 primary squamous cell carcinoma (SCC) and 8 primary adenocarcinoma (ADC)], 6 tonsil, and 6 placenta samples. Unless otherwise stated, microtomy was performed immediately prior to experiment initiation. Normal storage conditions refer to a monitored and controlled laboratory environment with a relative humidity range of 14.4–80.5% (average 46.8%) and a temperature range of 20.1–31.0 °C (average 21.6 °C) where tissue sections were not exposed to direct light.

### Immunohistochemistry

Serial sections were cut at 4-µm thickness and allowed to dry at room temperature (RT) overnight. IHC staining for PD-L1 was performed using four different anti-PD-L1 clones: PD-L1 IHC 22C3 pharmDx (Agilent; Santa Clara, CA, USA) and PD-L1 IHC 28-8 pharmDx (Agilent) per manufacturers guidelines; [10, 11] Cell Signaling Technology (Danvers, MA, USA) PD-L1 E1L3N, catalog #13684 (5.4 μg/mL) and Abcam (Cambridge, MA, USA) PD-L1 SP142, catalog #228462 (0.44 μg/mL) as laboratory developed tests using EnVision^TM^ FLEX detection (High pH) on the Autostainer Link 48 (Agilent). Immunostaining for pan-cytokeratin (pan-CK) was assessed as a control using an antihuman Cytokeratin, clone AE1/AE3, ready to use (Agilent; Catalog #IR053). A representative section from each block was stained for hematoxylin and eosin (H&E) on day 0 of each experiment. All stained tissue sections were scanned on an Aperio ScanScope AT Slide Imager (Leica Biosystems; Buffalo Grove, IL, USA) at ×40 magnification, and images viewed on Aperio ImageScope (v12.3.2) [28].

### Assessment of immunostaining

Expression of PD-L1 was assessed by pathologists trained and experienced in its interpretation according to interpretation guides where appropriate for NSCLC and gastric specimens [10–12, 29, 30]. For the 22C3, 28-8, and E1L3N anti-PD-L1 clones, a tumor proportion score (TPS) was calculated from the number of PD-L1 positive TCs as a proportion of all TCs and expressed as a percentage. Interpretation of the SP142 anti-PD-L1 antibody clone involved both TC and immune cell (IC) scoring, and was expressed as both a TPS and TC/IC score according to the established interpretation guidelines [12]. Gastric carcinoma specimens were assigned a combined positive score (CPS) that counts positive tumor and relevant ICs [30]. All specimens had PD-L1 and CK immunostaining assessed using the Aperio ImageScope integrated image analysis ‘Positive Pixel Count v9’ [31] algorithm to give an objective measurement of expression. The number of positive pixels taken as a proportion of the total number of tissue pixels was used to define the positivity score and given as either absolute values (positivity index) or as a percentage change relative to the corresponding sample at day 0 (positivity %).

### Accelerated degradation of unstained sections

Unstained sections of tissue were placed in a custom-built acceleration chamber contained within an incubator (Panasonic MIR-154-PA, Seacaucus, NJ, USA) without direct light exposure where humidity, oxygen concentration, and temperature could be regulated and measured. Environmental conditions of 37 °C, 100% oxygen, and humidity of ~80% (range 75–85%) were used as baseline parameters to achieve accelerated loss of detectable antigen as measured by IHC. Oxygen concentration was maintained and monitored using an oxygen meter [Apogee Instruments Oxygen Meter (MO-200); Logan, UT, USA] and humidity levels were measured using a Lockdown Hygrometer (Lockdown Vault Accessories; Columbia, MO, USA). Sections stored within the incubator were removed at appropriate time points for IHC staining alongside control sections stored in normal archival conditions (RT, atmospheric oxygen, and humidity). Repeat experiments of environmental effect on antigen degradation involved the change of these parameters individually. Experiments exploring the use of desiccant to protect against chamber conditions involved comparing antigen expression in sections placed within the chamber in a closed box, sealed in a protective bag (Minigrip Commercial LLC UV Protection Bag; Alpharetta, GA, USA) with desiccant (Fisherbrand Humidity Sponge Desiccant; Lenexa, KS, USA) and a humidity indicator card (WiseSorbent Technology (Marlton, NJ, USA)), to sections placed within the chamber without additional protection, as well as to sections stored under normal archival conditions.

### Peptide standards for MS

Synthetic, isotopically labeled PD-L1 peptide standards LQDAGVYR and AEVIWTSSDHQVLSGK containing U-^13^C_6_, U-^15^N_4_-arginine, or U-^13^C_6_, U-^15^N_2_-lysine at the C-termini and unlabeled peptide standards were purchased from New England Peptide (Gardner, MA, USA). Isotope labeled peptides were of greater than 99% and 95% isotopic and chemical purity, respectively; absolute concentration was determined by amino acid analysis.

### MS analyses

PD-L1 was analyzed by targeted MS as described previously [27] with the following modifications. To establish elution of oxidized tryptophan forms of the AEVIWTSSDHQVLSGK peptide, an aliquot of the peptide standard was treated with 1.5% hydrogen peroxide at RT for 1 min and then evaporated to dryness under vacuum. This standard contained a mixture of unoxidized AEVIWTSSDHQVLSGK and the tryptophan oxidation products AEVI[W + 4]TSSDHQVLSGK (kynurenine form) and AEVI[W + 16]TSSDHQVLSGK (monooxygenated form). Each sample analysis began with 100 µg tissue protein and after digestion was spiked with 50 fmol each of the labeled LQDAGVYR standard and the AEVIWTSSDHQVLSGK and oxidation product mixture. Tryptic peptide digests were fractionated by basic reverse phase liquid chromatography with disposable spin columns (Pierce™ High pH Reversed-Phase Peptide Fractionation Kit, Thermo Scientific, Rockford, IL, USA) according to the manufacturer’s instructions.

Targeted MS analyses were performed on an Orbitrap Fusion Lumos Tribrid™ instrument (Thermo Scientific, Bremen, Germany) equipped with an Easy nLC™ 1200 liquid chromatograph and a Nanospray Flex™ ion source (Thermo Scientific, San Jose, CA). Reverse phase liquid chromatography was done with a PepMap RSLC C18-3 micron column, 75 μm × 15 cm, eluted at 250 nL/min with a mobile phase gradient consisting of solvent A (0.1% aqueous formic acid) and solvent B [0.1% formic acid in water/acetonitrile (1:4, v/v)]. The mobile phase was initially 5% B and then programmed to 20% B over 18 min, to 35% B over 14 min and finally to 95% B over 5 min before recycling to starting composition. Targeted MS analysis was done by parallel reaction monitoring on the Lumos. The acquisition method consisted of a full scan selected ion monitoring event followed by targeted MS2 scans as triggered by a scheduled inclusion list, with a 5 min retention time window containing the precursor *m/z* values. Retention times were determined from prior analyses of synthetic peptide standards. The MS1 scan was collected at a resolution of 30,000, an automatic gain control (AGC) target value of 5e4, and a scan range from *m/z* 350 to 1000. MS1 data were recorded in profile mode. The MS1 scan was followed by targeted MS2 collision induced dissociation scans at a resolution of 30,000, an AGC target value of 5e4, 1.6 *m/z* isolation window, activation Q of 0.25 and an optimized collision energy for each target of 30%. MS2 data were recorded in profile mode. Parallel reaction monitoring transitions were extracted from raw datafiles and analyzed with Skyline [32]. Peptide peak areas were calculated as the sum of three most abundant transitions. Peptide abundance was calculated from the ratio of peak area for the unlabeled endogenous peptide to the labeled internal standard.

Global proteome analyses were performed on unfractionated tryptic digests of the same samples with the same MS instrument, chromatography system and source. Reverse phase liquid chromatography was performed with a PepMap RSLC C18-3 micron column, 75 μm × 30 cm, eluted at 250 nL/min with a mobile phase gradient consisting of solvent A (0.1% aqueous formic acid) and solvent B [0.1% formic acid in water/acetonitrile (1:4, v/v)]. The mobile phase was initially 6% B and then programmed to 27% B over 27 min, to 40% B over 40 min, and finally to 95% B over 8 min before recycling to starting composition. An MS1 scan was collected at a resolution of 120,000, an AGC target value of 4e5, a maximum injection time of 50 ms, and a scan range from *m/z* 375 to 1500. MS1 data were recorded in profile mode. MS2 high-energy collision induced dissociation scans were acquired at an AGC target value of 1e4, a maximum injection time of 35 ms, and with an isolation window of 1.2 *m/z*. Tandem MS scans were acquired as centroided data. Peptide sequence identification from tandem mass spectra was done as described previously [33], except that the search engine for peptide-spectrum matches was MS-GF+ [34]. Peptide-spectrum matches were performed with an FDR threshold of 1%, and required at least two distinct peptide identifications per protein identification. Protein abundance differences were calculated from spectral counts.

### Statistical analysis

Statistical analysis was performed using IBM SPSS statistics software, version 25 (IBM Corp, Armonk, NY, USA). Comparisons of multiple groups over time were performed using repeated measures ANOVA with Bonferroni correction and Tukey’s post-hoc analysis. Comparisons of two groups at a single point were performed using independent samples or paired Student’s *t* test as appropriate. Relationships between variables were assessed using Pearson’s correlation and, if appropriate, linear regression. Analysis of the effect of incubation conditions on methionine oxidized peptides compared the baseline condition and day 28 accelerated degradation condition and used peptide count data for peptides with at least ten spectral counts using Fisher’s exact test (two-sided). The Wilcoxon Signed Rank test was used to determine significance in the number of oxidized peptides between the baseline and day 28 conditions. Analyses of spectral counts were performed using R software for statistical computing (version 3.4.3) and all significances were taken as *p* < 0.05.

## Results

### Natural timeline of PD-L1 loss under normal storage conditions

**Fig. 1:**
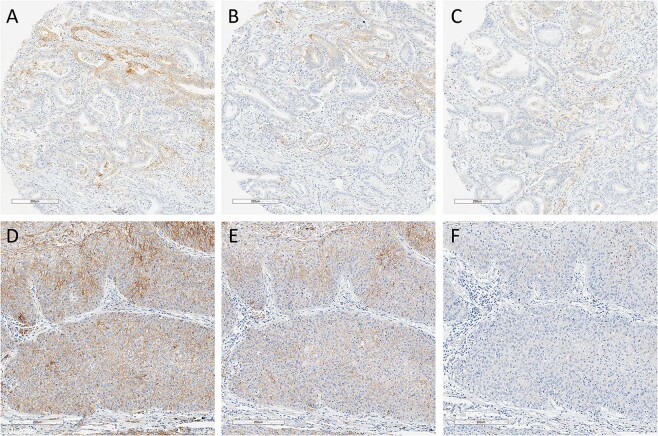
PD-L1 expression in aged tissue and tissue under accelerated conditions. Representative PD-L1 expression assessed by E1L3N IHC in FFPE gastric carcinoma under normal atmospheric conditions (**a**–**c**) and in NSCLC under acceleration conditions (**d**–**f**). **a** Day 0, **b** 4.5 months, **c** 24 months; **d** Day 0, **e** Day 9, **f** Day 28. PD-L1 programmed-death-ligand-1, IHC immunohistochemistry, FFPE formalin-fixed, paraffin embedded, NSCLC non-small cell lung cancer.

To confirm the PD-L1 immunoreactivity loss in stored tissue sections that has been reported in literature could be reproduced in our laboratory, TMA sections containing 35 gastric carcinomas stored in archive over 24 months under normal ambient conditions were stained for PD-L1 using E1L3N and SP142 clones. Positive pixel count scoring (positivity) demonstrated that E1L3N detected PD-L1 with a greater sensitivity than SP142 (average positivity score 0.197 vs 0.128; *p* < 0.001), but both clones showed significant loss of PD-L1 expression over time as expected (average positivity score E1L3N at 4.5 months and 24 months 0.197 vs 0.107; *p* = 0.05, 0.197 vs 0.070; *p* < 0.001; SP142 at 4.5 months and 24 months 0.128 vs 0.075; *p* < 0.001, 0.128 vs 0.074; *p* < 0.001). Examples are shown in Fig. 1a–c. CPS assessments for the gastric cancer TMAs were higher on average for E1L3N than for SP142 (CPS 40 vs 30; *p* < 0.05). Clinically relevant loss of CPS (from ≥1% to <1%) was seen by 4.5 months for both clones (E1L3N 13% of cases, SP142 20% of cases) with further loss by 24 months (E1L3N 33% of cases, SP142 37% of cases).

### PD-L1 loss under accelerated conditions

To determine whether the natural loss of immunoreactivity could be reproduced in an accelerated fashion, tissues were subjected to controlled environmental stress. Storing unstained sections of the NSCLCs in the acceleration chamber at 100% oxygen, 37 °C, and 80% humidity resulted in repeatable stepwise loss of PD-L1 expression over 28 days comparable with loss, in effect, seen over 24 months in ambient conditions (Fig. 1d–f).

The NSCLC tissues were stained and assessed for PD-L1 expression using multiple antibody clones. Image analysis of these demonstrated day 0 PD-L1 expression was broadly equivalent for 22C3 and 28-8, with a slightly increased average expression for E1L3N, and markedly lower average expression for SP142. For the 22C3, 28-8, and E1L3N clones a stepwise loss of PD-L1 expression is seen over 28 days within the acceleration chamber. The low immunostaining by SP142 at day 0 resulted in minimal further detection of expression decrease across NSCLC specimens thereafter. (Supplementary Fig. S1).

**Fig. 2:**
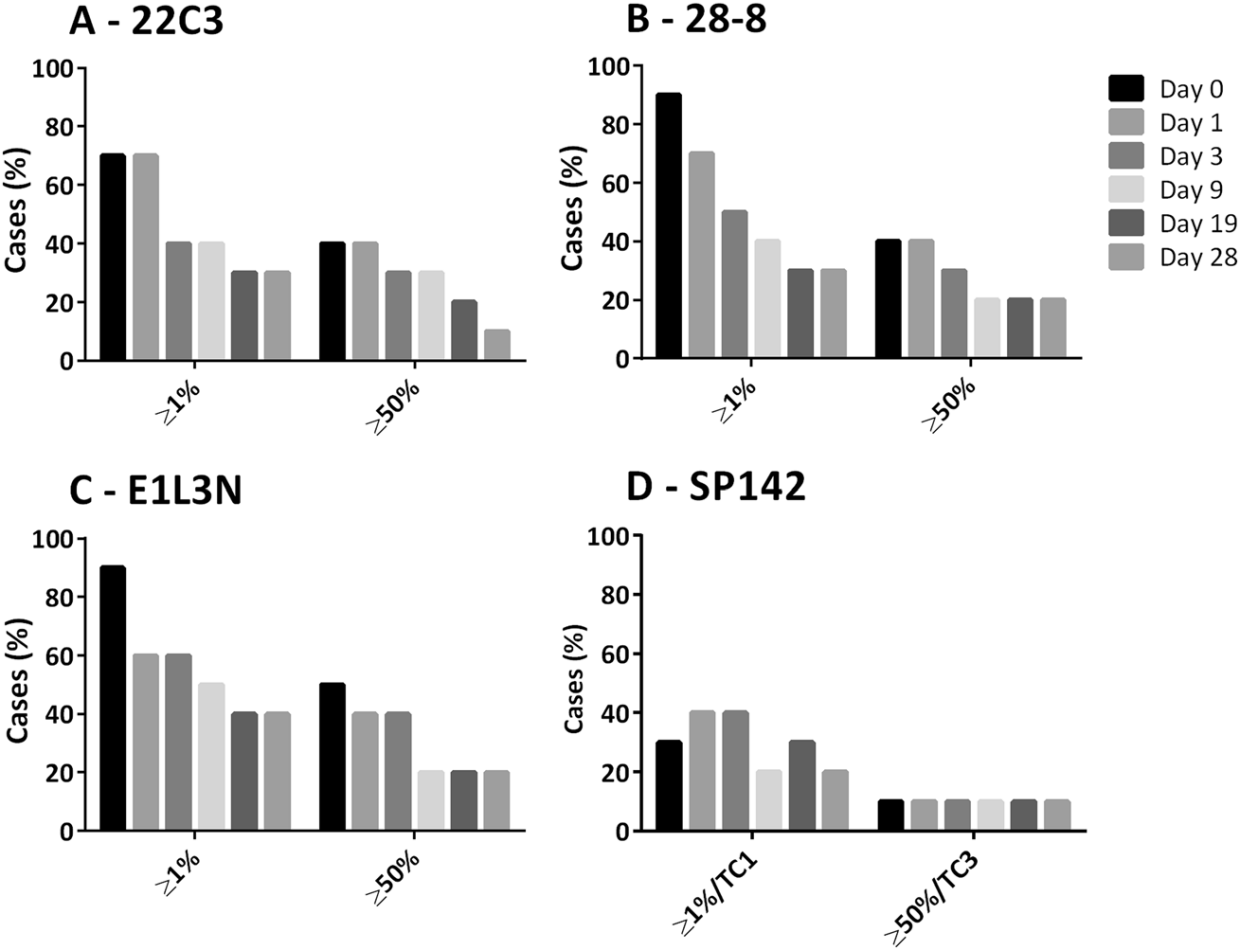
PD-L1 by clinical cutoffs in FFPE NSCLC sections over time in the acceleration chamber with conditions of 100% oxygen, 80% humidity, and 37 °C for 22C3, 28-8, E1L3N, and SP142 PD-L1 clones. Bars represent number of cases in series with PD-L1 expression equal or above TPS clinical cutoff thresholds. PD-L1 programmed-death-ligand-1, TPS tumor proportion score, TC tumor cell, NSCLC non-small cell lung cancer, FFPE formalin-fixed, paraffin embedded.

PD-L1 expression by TPS found similar numbers of positive (TPS ≥ 1%) and strongly positive (TPS ≥ 50%) cases when assessed with the 22C3 (seven and four cases, respectively), 28-8 (nine and four cases, respectively), and E1L3N (9 and 5 cases, respectively) clones, but fewer positive and strongly positive cases for SP142 (three and one, respectively). Loss of PD-L1 TPS in NSCLC sections to levels below these prescribing guideline cutoffs occurred for all clones at varying time points, with over half of cases changing from diagnostically positive to diagnostically negative by day 19 for 22C3 (TPS ≥ 1% and ≥50%), day 9 for 28-8 (TPS ≥ 1% and ≥50%), and day 9 for positive and day 19 for strongly positive for E1L3N (Fig. 2).

### Effect of environmental conditions on immunoreactivity loss by IHC assessment

In order to further understand the relative contribution of major environmental conditions on tissue immunoreactivity, the effect of oxygen, humidity, and temperature on PD-L1 (E1L3N) and pan-CK (AE1/AE3) IHC expression was assessed on tonsil and placenta tissue sections stored in the acceleration chamber set to baseline conditions of 100% oxygen, 37 °C, and 80% humidity over 28 days. By day 28 significant loss of PD-L1 and pan-CK positivity was seen in both tissues (average positivity day 0 vs day 28: PD-L1 in placenta 0.528 vs 0.088 (100% vs 17%); *p* < 0.001, tonsil 0.123 vs 0.018 (100% vs 15%) *p* < 0.001; CK in placenta, 0.626 vs 0.259 (100% vs 41%); *p* = 0.05, tonsil 0.319 vs 0.219 (100% vs 69%); *p* < 0.05). Control slides kept at normal ambient conditions had no significant loss of either PD-L1 or pan-CK expression by day 28 for both placenta and tonsil tissue.

**Fig. 3:**
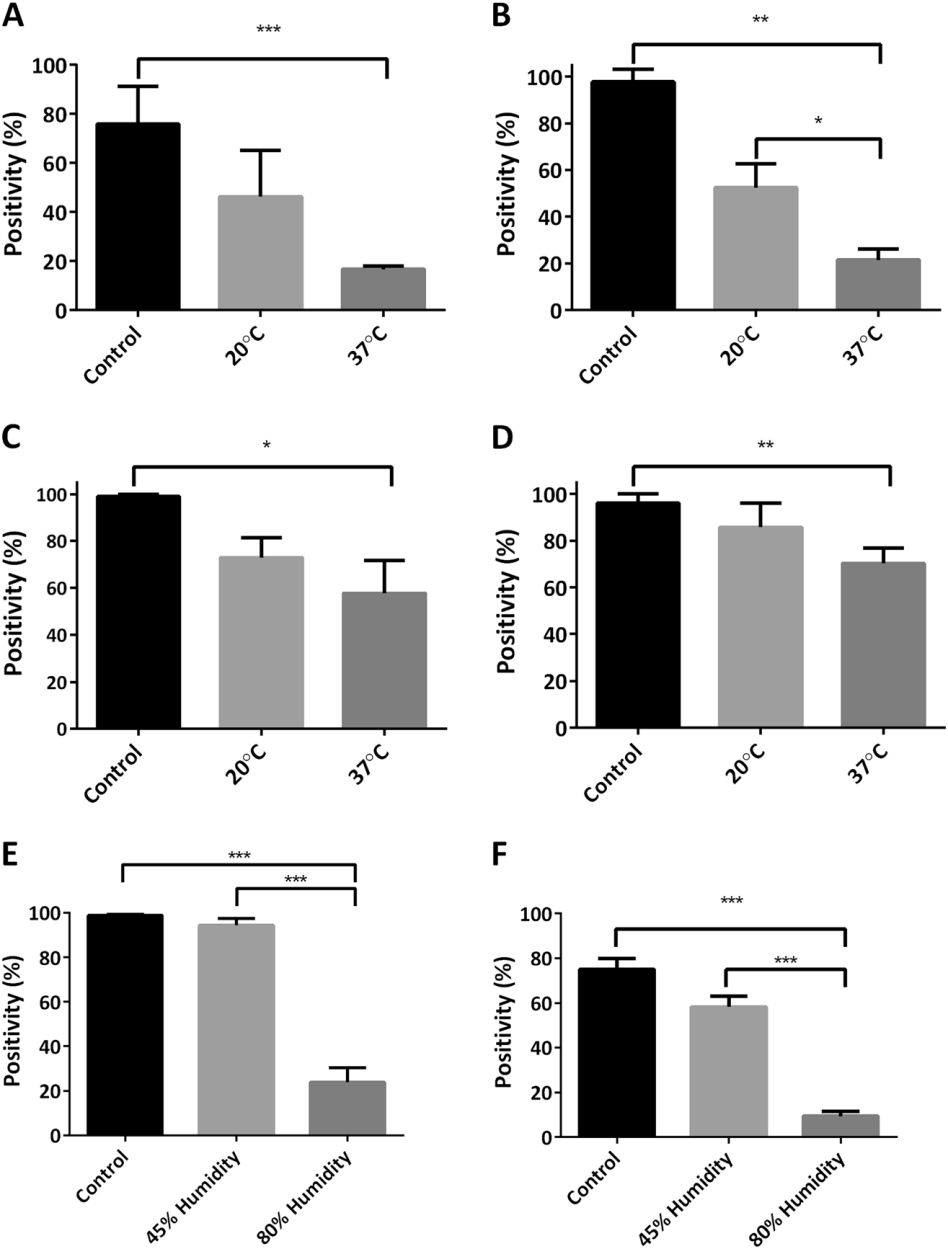
PD-L1 expression in NSCLC under varying accelerated conditions. Placenta and tonsil FFPE sections incubated in the acceleration chamber under different environmental conditions at day 28; **a**–**d**: 100% oxygen and 80% humidity at either 20 °C or 37 °C, then stained for PD-L1 (E1L3N) or pan-CK (AE1/AE3): **a** Placenta PD-L1, **b** Tonsil PD-L1, **c** Placenta pan-CK, **d** Tonsil pan-CK. 100% oxygen and 37 °C at either 45% or 80% humidity at day 28, **e** Placenta PD-L1, **f** Tonsil PD-L1. Control conditions: 20 °C, atmospheric humidity and oxygen. Bar represents mean ± SEM. *p < 0.05, **p < 0.01, ***p < 0.001. PD-L1 programmed-death-ligand-1, CK cytokeratin, FFPE formalin-fixed, paraffin embedded.

Changing the temperature in the acceleration chamber had a significant impact on the rate of IHC signal loss. Increasing the temperature to 60 °C (in the context of elevated oxygen and humidity) resulted in extremely rapid loss of PD-L1 expression: (day 7 PD-L1 expression reduced to 8% and 3% positivity in placenta and tonsil respectively; graph not shown). Conversely, decreasing the temperature to 20 °C reduced immunoreactivity loss, resulting in no statistically significant loss of PD-L1 in placenta tissue by day 28 (average positivity, 0.538 vs 0.257 (100% vs 48%); *p* = 0.174) or pan-CK in both placenta and tonsil tissue (average positivity, placenta 0.615 vs 0.215 (100% vs 35%); *p* = 0.284, tonsil 0.293 vs 0.247 (100% vs 84%); *p* = 0.423) with a significant reduction seen only for PD-L1 expression in tonsil by day 28 (average positivity 0.135 vs 0.07 (100% vs 52%); *p* < 0.05) though this was significantly less than the loss seen under 37 °C conditions (average positivity of PD-L1 in tonsil by day 28, 20 °C vs 37 °C: 0.07 vs 0.02 (52% vs 14%); *p* < 0.05), results summarized in Fig. 3a–d.

Reducing humidity had a significant impact on the rate of IHC signal loss. Decreasing humidity to 45% (in the context of elevated oxygen and temperature) resulted in no significant loss of PD-L1 expression in placenta at 39 weeks (0.639 vs 0.408 (100% vs 64%); *p* = 0.201) and required 28 weeks for a significant loss in tonsil to occur (0.183 vs 0.021 (100% vs 10%); *p* < 0.05). The rate of PD-L1 expression loss in reduced humidity conditions was slowed to the extent that both placenta and tonsil tissue demonstrated loss by 39 weeks at 45% humidity similar to, or less than, 1 week at 80% humidity (average positivity for placenta 0.41 vs 0.17 (64% vs 27%); *p* = 0.13, and tonsil 0.018 vs 0.019 (10% vs 10%); *p* = 0.93). Average positivity of PD-L1 in placenta and tonsil at 28 days under 45% and 80% humidity are shown in Fig. 3e–f.

Reducing oxygen levels in the incubation chamber to 20% (in the context of elevated temperature and humidity) had no significant effect on the rate or total quantity of either PD-L1 or pan-CK expression in either placenta or tonsil tissue (day 28 positivity, 100% vs 20% oxygen: PD-L1, placenta 0.074 vs 0.08 (14% vs 16%); *p* = 0.918; tonsil 0.0079 vs 0.016 (7% vs 14%); *p* = 0.937; pan-CK, placenta 0.43 vs 0.45 (68% vs 71%) *p* = 0.918; tonsil 0.21 vs 0.22 (61% vs 64%); *p* = 0.937). Results shown in Supplementary Fig. S2.

### Effect of desiccant on preventing immunoreactivity loss

Because humidity was determined to be a major factor in loss of tissue immunoreactivity, the effect of storing slides with or without desiccant in the acceleration chamber set to baseline conditions of 100% oxygen, 37 °C and 80% humidity over 28 days was examined. This was accomplished by measuring the expression of PD-L1 (E1L3N) and pan-CK IHC using positive pixel count scoring (positivity) on placenta and tonsil tissue sections stored over 28 days.

Upon removal of slides from the sealed container stored within the acceleration chamber, the humidity level was recorded as <30% using the enclosed indicator card. Slides stored with desiccant showed no significant loss of either PD-L1 or pan-CK expression in any tissue at day 28 (Average positivity day 0 vs day 28: PD-L1 in placenta 0.57 vs 0.53 (100% vs 93%); *p* = 0.083, tonsil 0.088 vs 0.073 (100% vs 83%); *p* = 0.555, pan-CK in placenta, 0.74 vs 0.72 (100% vs 97%); *p* = 0.311, tonsil 0.33 vs 0.30 (100% vs 91%); *p* = 0.185).

**Fig. 4:**
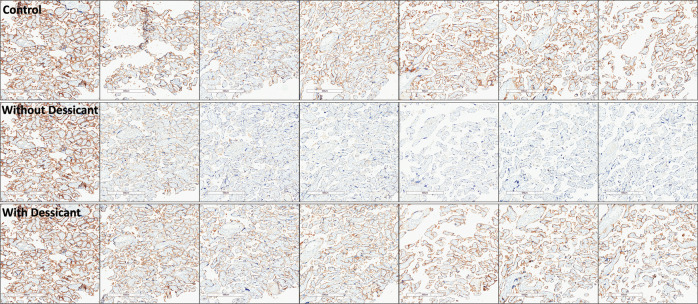
PD-L1 expression by E1L3N in FFPE placenta sections at days 0, 1, 3, 7, 14, 21, and 28. The first row shows tissue sections stored under normal atmospheric conditions, the second and third row show tissue sections within an incubator at 100% oxygen, 37 °C, and 80% humidity without (second row) and with (third row) desiccant. PD-L1 programmed-death-ligand-1, FFPE formalin-fixed, paraffin embedded.

**Fig. 5:**
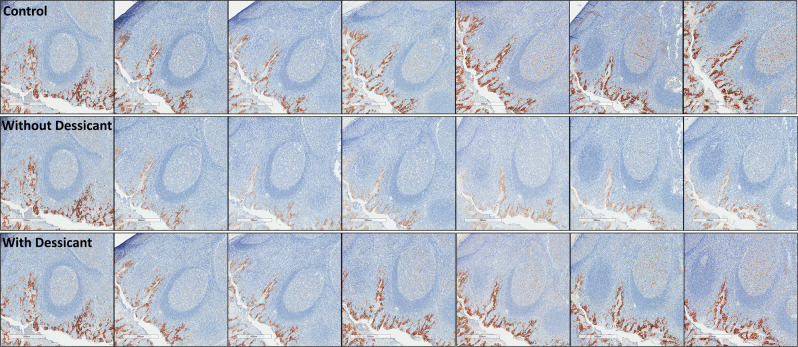
PD-L1 expression by E1L3N in FFPE tonsil sections at days 0, 1, 3, 7, 14, 21, and 28. The first row shows tissue sections stored under normal ambient conditions, the second and third row shows tissue sections within an incubator at 100% oxygen, 37 °C and 80% humidity without (second row) and with (third row) desiccant. PD-L1 programmed-death-ligand-1, FFPE formalin-fixed, paraffin embedded.

Qualitative assessment of sections showed slides stored with desiccant demonstrate expression loss similar to sections stored under normal atmospheric conditions, with appreciable loss of PD-L1 expression in sections stored without desiccant (Figs. 4 and  5). To a lesser extent, loss of pan-CK was observed (Supplementary Fig. S3a, b). Significant loss of PD-L1 immunoreactivity was seen for placenta tissue stored within the acceleration chamber, with appreciable, but nonstatistically significant loss demonstrated in tonsil tissue. (Supplementary Fig. S4)

### PD-L1 immunoreactivity loss on specific cell types

Tonsil tissue stained for PD-L1 with E1L3N stored in the acceleration chamber with conditions of 100% oxygen, 80% humidity and 37 °C over 28 days was assessed for expression loss difference between crypt epithelium cells and ICs associated with germinal centers. Day 0 sections showed strong staining within the crypt epithelium, and weaker staining within the geminal centers, in keeping with known PD-L1 expression variation between these cell types [29]. Loss is seen in a stepwise fashion over time, with no difference in the rate of loss seen between the crypt epithelia and the germinal centers (Supplementary Fig. S5). Due to the weaker immunoreactivity associated with these ICs at baseline; however, signal loss in this cell type was appreciated earlier in the time course and was particularly noticeable when scanning tissues at lower magnification. This observation suggests clinical scoring guidelines that rely upon characterization of weaker staining cells may be impacted differentially by storage conditions.

### Targeted MS analyses of PD-L1

To test the hypothesis that peptide oxidation is a major contributor to loss of PD-L1 immunoreactivity in stored tissues, targeted MS analyses of PD-L1 peptides were performed to measure PD-L1 protein abundance in specimens stored in normal atmospheric conditions or within the acceleration chamber with conditions of 100% oxygen, 37 °C, and 80% humidity. Two PD-L1 peptides were analyzed, LQDAGVYR and AEVIWTSSDHQVLSGK, both of which are in the extracellular domain of PD-L1. Whereas LQDAGVYR contains no easily oxidizable residues, AEVIWTSSDHQVLSGK contains an oxidizable tryptophan (W) and lies within the recognition sequence for the 22C3 antibody [35]. It has been shown previously that measurement of the LQDAGVYR peptide in FFPE melanoma specimens yielded PD-L1 abundance comparisons similar to those obtained by IHC with the E1L3N antibody [27]. Thus, for these MS analyses, the LQDAGVYR peptide was measured to quantify the abundance of PD-L1 protein, whereas the AEVIWTSSDHQVLSGK was measured as an oxidation-sensitive site. Moreover, we monitored W oxidation products of AEVIWTSSDHQVLSGK to detect oxidative changes that could affect the 22C3 recognition site. Representative MS traces for the peptides and the oxidation product are shown in Supplementary Fig. S6.

**Fig. 6:**
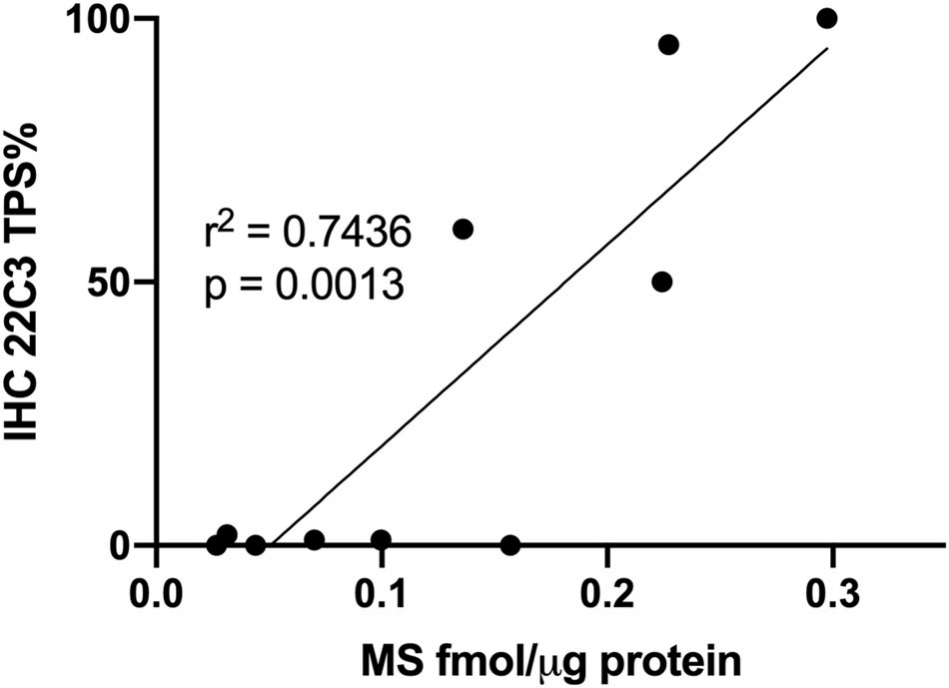
Correlation of PD-L1 protein expression by immunohistochemistry (by TPS) with PD-L1 abundance measured by MS in FFPE sections prior to incubation in the accelerated loss chamber. PD-L1 programmed-death-ligand-1, TPS tumor proportion score, MS mass spectrometry, FFPE formalin-fixed, paraffin embedded.

**Fig. 7:**
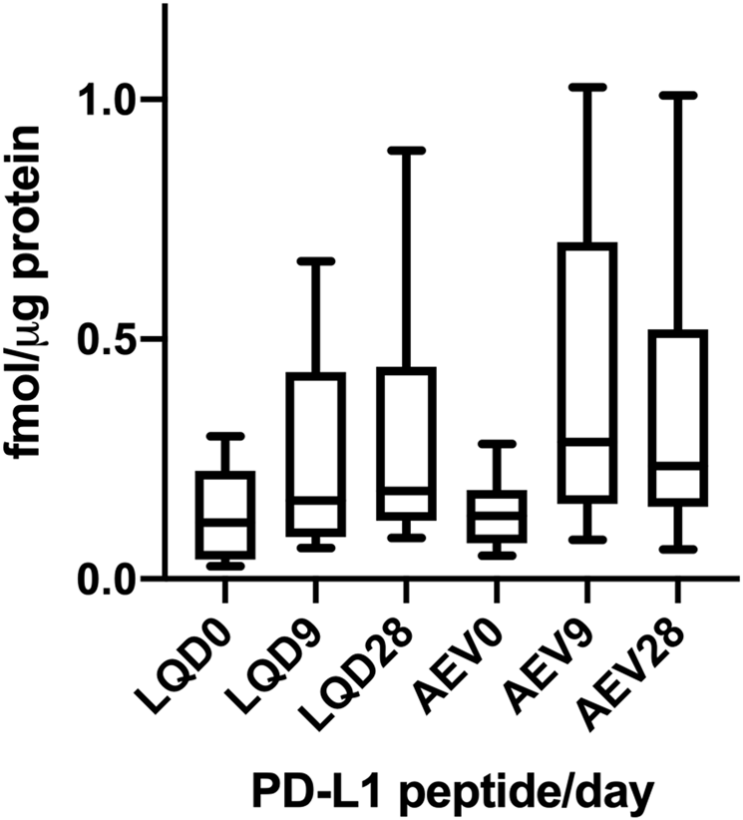
MS quantitation of PD-L1 peptides LQDAGVYR (LQD) and AEVIWTSSDHQVLSGK (AEV) in FFPE sections incubated in accelerated loss chamber at baseline (0), 9 and 28 days of incubation. MS mass spectrometry, PD-L1 programmed-death-ligand-1, FFPE formalin-fixed, paraffin embedded.

MS measurements of PD-L1 peptide LQDAGVYR at baseline yielded protein abundance estimates concordant with IHC with the 22C3 antibody (*r*^2^ = 0.744, *p* = 0.001) (Fig. 6). MS analyses of LQDAGVYR and AEVIWTSSDHQVLSGK at baseline and at 9 and 28 days of the acceleration protocol indicated that PD-L1 levels were not decreased in the NSCLC samples, in contrast to IHC measurements (Fig. 7). We noted that variation in measured PD-L1 abundance appeared to be increased in the 9 and 28 day samples. This effect may reflect inconsistent recovery of the labeled standard peptides in the samples subjected to the acceleration protocol. Nevertheless, comparison of the MS peak areas for the endogenous PD-L1 peptide at baseline, 9 and 28 days did not reveal any apparent loss of PD-L1 protein with incubation time (Supplementary Fig. S7).

Oxidation of PD-L1 tryptophan residue in AEVIWTSSDHQVLSGK would occur within the 22C3 recognition sequence and might affect antibody binding. Hydrogen peroxide treatment of the AEVIWTSSDHQVLSGK labeled standard yielded the kynurenine form AEVIW[+4]TSSDHQVLSGK as the predominant product. In MS analyses of the 28 day incubated samples, we detected the AEVIW[+4]TSSDHQVLSGK labeled standard, but not any oxidized peptide from the NSCLC samples (Supplementary Fig. S6).

**Fig. 8:**
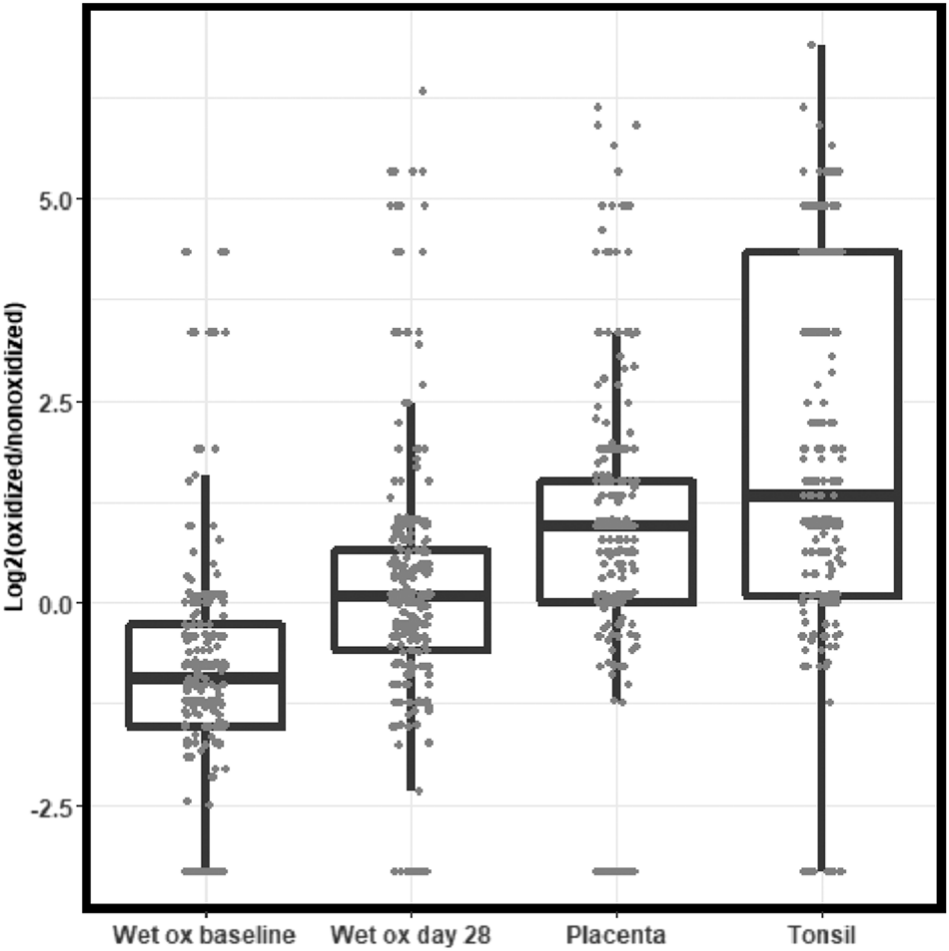
Global proteome analyses to assess proteome-wide oxidation under baseline (wet ox baseline) and acceleration conditions (wet ox day 28) compared with samples of naturally aged placenta and tonsil tissue stored under normal ambient conditions. The plotted values are log2 ratios of numbers of MS/MS spectra corresponding to oxidized and unoxidized methionine-containing peptides. Higher log2 ratios correspond to greater extent of proteome oxidation.

To further test the hypothesis that the acceleration chamber produced oxidation of proteins in FFPE sections that interferes with immunoreactivity by IHC, we performed global MS analyses of four samples each of the baseline and day 28 samples from the acceleration chamber experiment, as well as two samples each from placenta and tonsil stored as unstained sections under ambient conditions for 2 years. We extracted data for a set of 274 methionine-containing peptides that were found in all samples in both reduced and oxidized forms and compared abundances as the log2 ratio of spectral counts for both forms (Fig. 8). Shifts in distributions to values greater than 1 indicated increased oxidation. Though the distributions overlapped substantially, the effect of 28 day wet oxidation was significant (Dunn’s test, adj. *p* = 9 × 10^−14^). The distributions for ambient stored placenta and tonsil were similar to the 28 day wet oxidized NSCLC from the acceleration chamber [Dunn’s test (adj. *p* = 1 × 10^−34^ (tonsil vs wet oxidation baseline)); adj *p* = 8 × 10^−58^ (placenta vs. wet oxidation baseline)]. Although these global data indicate the presence of statistically significant oxidation, the distributions clearly indicate a modest overall degree of oxidation that could not account for the near-complete loss of PD-L1 IHC staining observed.

## Discussion

The loss of immunostaining in archived FFPE tissue is a well-known but poorly understood phenomenon [22]. The role of IHC in the analysis of predictive biomarkers has increased the precision required for the use of immunostaining techniques, and the challenges of these are well illustrated in the difficulties of using PD-L1 IHC as a predictive biomarker of response to anti-PD-1/PD-L1 immune checkpoint inhibitors [7]. The apparent loss of PD-L1 expression in aged specimens has raised justifiable concerns about the use of older tissue blocks and sections. This concern has led to guidance to use new tissue blocks where possible and test sections as soon as they are prepared, with varied recommendations between both specific PD-L1 clones and tissue types [10–13, 36].

The loss of antigen immunoreactivity has been observed to vary between different antigens within and between tissue types, with no obviously consistent factors that may help predict the most sensitive antigens. Both polyclonal and monoclonal antibodies may be affected, and loss occurs in IHC assays that target the nucleus, cytoplasm, or membranes of cells. Specific IHC signal loss can vary between studies depending on factors such as fixation and the specific antibodies used [3, 16, 18–21, 37–39].

Mechanisms of antigen degradation have been explored previously, with a variety of potential factors thought to influence the loss, including: preanalytical variables, oxidation, humidity, and high temperature [16, 20, 22–24, 40, 41]. We demonstrated with MS that there is no detectable loss of the PD-L1 protein peptides measured in stored tissues, even when IHC demonstrated major reductions in quantity and intensity of immunoreactivity. Therefore, conformational changes to the antigens themselves are more likely to account for the reduction seen.

The role of oxidation in antigen degradation has been suggested previously [20, 40], and our global proteome analyses indicate that conditions that facilitate accelerated wet-air oxidation caused a statistically significant degree of oxidation that was similar to that seen in placenta and tonsil samples stored under ambient conditions for 2 years. However, this extent of oxidation was likely insufficient to account for the reduction in PD-L1 immunostaining by IHC either in the acceleration experiments, or under normal ambient storage conditions. Furthermore, we found no evidence that changing the storage conditions from low-to-high oxygen content affected PD-L1 or pan-CK detection by IHC to any significant degree.

Previous work suggested that humidity or the fixation process can have an effect on antigen degradation [23, 42–44]. Formaldehyde fixation results in multiple crosslinking interactions that can involve proteins or DNA and chromatin [44–46], but aldehyde induced crosslinks are susceptible to spontaneous hydrolysis, a process catalyzed by higher temperature [47, 48]. Our findings are consistent with previous reports that the presence of water and high temperature are a major cause of antigen loss in FFPE tissue [16, 23, 49]. Therefore, one mechanism by which antigen expression may be lost is heat catalyzed hydrolysis of susceptible protein–protein crosslinks, resulting in a change in crosslinked protein structures and loss of discontinuous epitope sites or masking of linear sites.

The crosslinking process increases accessibility to some antigens and renders others inaccessible through masking of epitopes [42, 50]. The mechanism of crosslinking and antigen masking is not fully characterized, but the masking of antigens in FFPE tissue is more likely with specific amino acid sequences [51, 52], and, importantly, discontinuous epitopes [52, 53] that are also particularly susceptible to loss in high temperatures [43]. PD-L1 expression loss in archived tissue has been seen in previous studies [1, 8, 9, 54], and our results are aligned. Anti-PD-L1 IHC clones detect a variety of epitope regions, many of which are believed to be discontinuous (28-8, SP263, and SP142) [37, 55, 56], possibly explaining why PD-L1 IHC is particularly sensitive to loss of immunoreactivity. An alternative explanation for this observation includes the possibility that the extracellular epitopes recognized by 22C3 and 28-8 are particularly accessible, and therefore are more susceptible to environmental humidity during tissue storage.

Tissue processing and antigen retrieval prior to IHC involves dehydration and a rehydration of tissue, which has been demonstrated to be a crucial step in achieving successful IHC assay outcomes [43, 57]. Interestingly, the effect observed with the acceleration chamber was prevented with the use of desiccant stored alongside the sections, to the extent that the minimal loss of immunoreactivity is similar to that in sections stored under normal ambient conditions. This suggests that humidity during storage is a major driving force behind immunoreactivity loss. This may be due to epitope conformational changes driven by hydration, which occurs over time during storage, but is not of the same nature as the rehydration of tissue that occurs during antigen retrieval immediately prior to immunohistochemical staining. The practical implications of this finding are significant: desiccant may provide an effective method of preventing antigen loss that could be immediately implemented into clinical research protocols involving the storage and transportation of tissue. This would provide an attractive alternative to other more complicated, time-consuming, and expensive methods of preventing loss such as microwave heating, recoating in paraffin wax, storage under vacuum, or the use of nitrogen chambers [14, 20, 22, 24, 58].

This study has limitations. While over a thousand tissue sections were included in the analysis, sample sizes were small for some conditions, perhaps accounting for nonstatistically significant trends in certain experiments. Positivity as defined by pixel counting was complemented by TPS/CPS scores in tumors to give clinical relevance, but the equivalent is not possible in placenta and tonsil; therefore, although significant loss can be quantitatively demonstrated with good reproducibility (Supplementary Figs. S8–S9, Supplementary Tables S1–S2), the point of clinically relevant loss does not translate for these tissues. Although PD-L1 expression by IHC did broadly correlate with MS findings, not all cases did, and these discrepancies could be accounted for by PD-L1 protein posttranslational modifications including glycosylation [27] which may not be detected by IHC but are detectable by MS. Labeled internal peptide standards used in the targeted analysis of PD-L1 indicated that peak areas were reduced in some, but not all, samples. While the cause of this is uncertain, we cannot rule out the possibility that the accelerated degradation conditions may have altered those specimens in a manner that reduced recovery of the labeled standards. Inspection of the MS peak areas for the endogenous PD-L1 peptides indicated no loss of protein during accelerated incubation (Supplementary Fig. S7). Finally, the acceleration incubator has demonstrated that select environmental conditions reproducibly affect the loss of IHC expression, but this has focused on PD-L1 and pan-CK in specific tissues. The application of this approach as a wider tool in understanding antigen loss under ambient conditions, and the optimal conditions to predict storage effect on novel biomarkers in development requires further study.

To our knowledge, this is the first study that has systematically used quantitative MS to characterize the impact of tissue storage using a model system under controlled conditions. We demonstrate that PD-L1 expression assessed by IHC with different antibody clones undergoes signal loss over time, and that this loss is largely accelerated by humidity and high temperature, rather than by environmental factors favoring oxidation. The use of an acceleration process to mimic the natural loss of antigenicity that occurs in naturally stored FFPE tissue sections may provide a platform by which novel biomarker robustness can be evaluated early in development. MS is a powerful technique that overcomes the limitations of studying protein expression in stored tissues sections by IHC alone. Moreover, MS may be ideally suited to analyze archival FFPE specimens and conduct hypothesis testing in regards to the abundance of protein drug targets with associated therapeutic outcomes.

## Supplementary information

Supplementary figures S1–S9 and supplementary tables S1–S2
