# Supplementary material for: Accelerated instability testing reveals quantitative mass spectrometry overcomes specimen storage limitations associated with PD-L1 immunohistochemistry
Source: Lab Invest. 2020 Jan 2;100(6):874–86. doi: 10.1038/s41374-019-0366-y (PMC7280177; doi:10.1038/s41374-019-0366-y)
Supplement: Supplementary file 1 — Supplementary figures S1–S9 and supplementary tables S1–S2 [file 41374_2019_366_MOESM1_ESM.pdf]

Supplementary figures S1-S9 and supplementary tables S1-S2 for "Accelerated Instability Testing Reveals Quantitative Mass Spectrometry Overcomes Specimen Storage Limitations Associated with PD-L1 Immunohistochemistry"

Supplementary figure S1

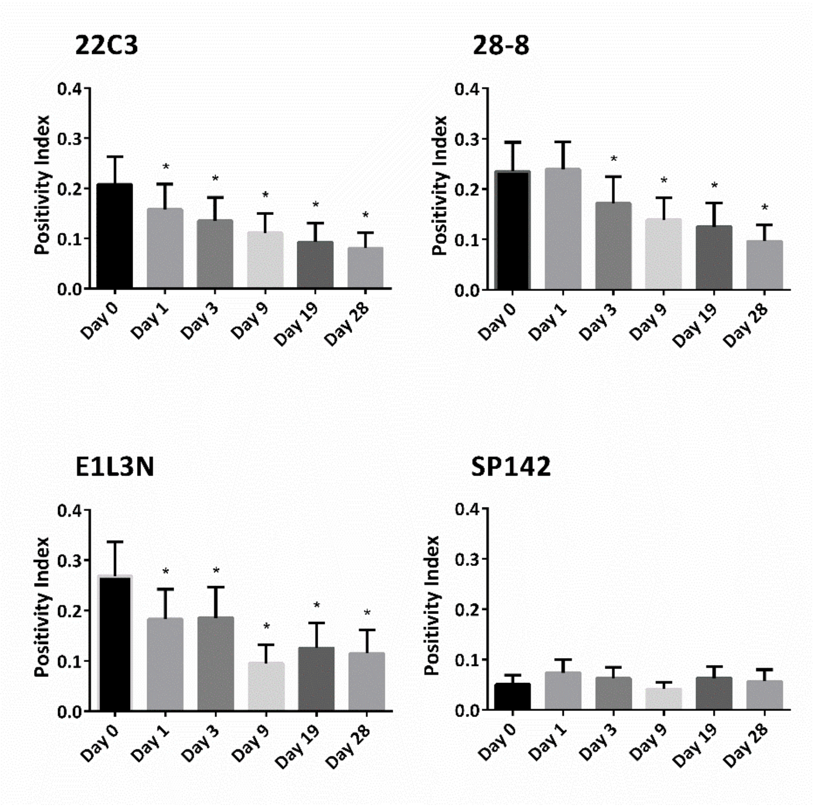

Fig S1. PD-L1 expression by positive pixel count in NSCLC sections over time in the acceleration chamber with conditions of 100% oxygen, 80% humidity, and 37°C for 22C3, 28-8, E1L3N, and SP142 PD-L1 clones. Significant reduction in PD-L1 expression is measured against day 0 values. Bars represent mean ± SEM. *$p<0.05$.

PD-L1, programmed-death ligand-1; NSCLC, non-small cell lung cancer.

Supplementary figure S2

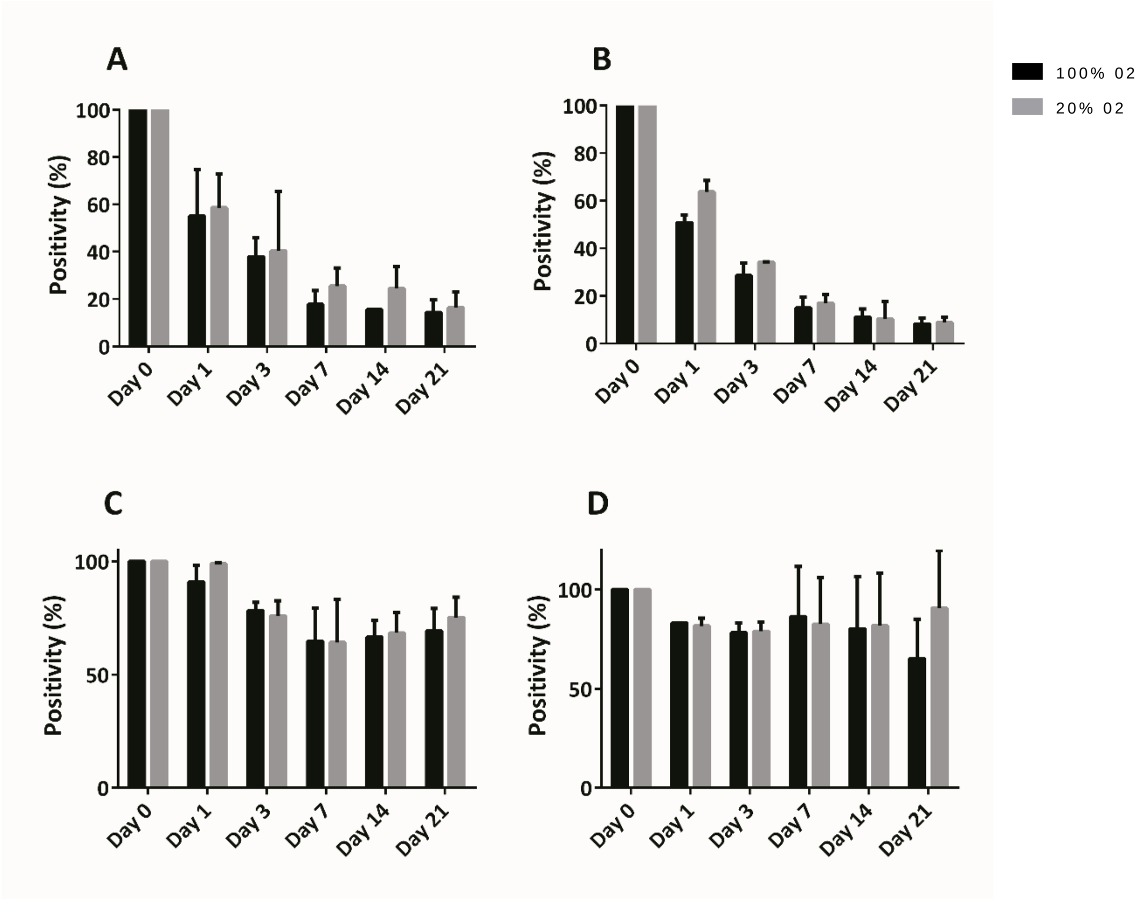

Fig S2. IHC for PD-L1 expression (E1L3N) (A and B) and pan-CK (AE1/AE3) (C and D) by positive pixel count in placenta (A/C) and tonsil (B/D) sections in the acceleration chamber with conditions of 80% humidity and 37°C at either 100% oxygen or 20% oxygen over time. No significant difference is seen between sections at high (100% oxygen) or low (20% oxygen) at any timepoint in any tissue for either PD-L1 or pan-CK. Bars represent mean ± SEM.
PD-L1, programmed-death ligand-1; CK, cytokeratin.

Supplementary figure S3a and S3b

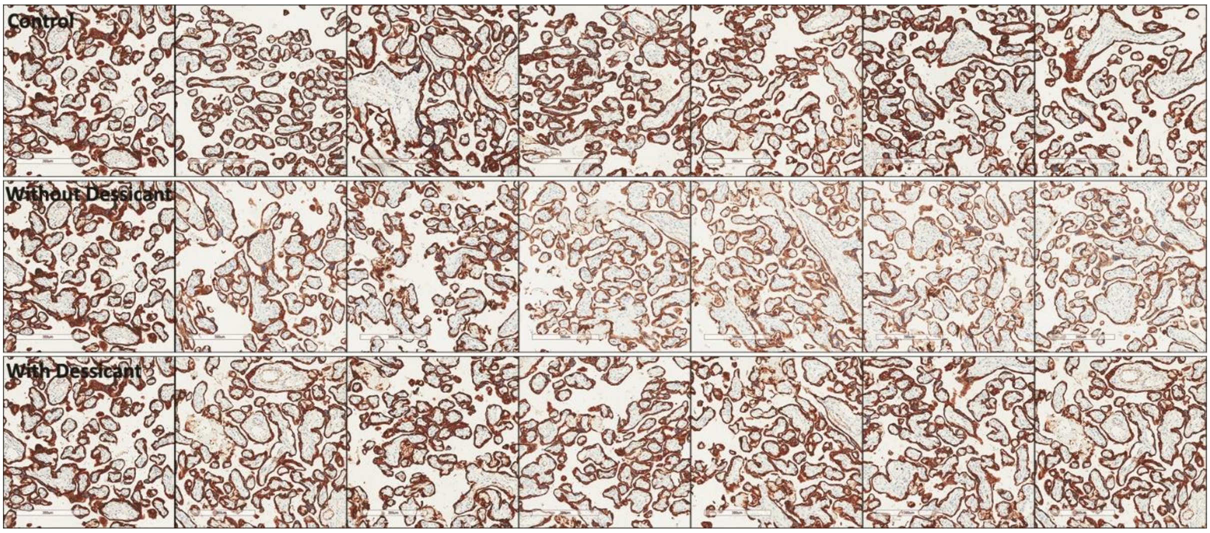

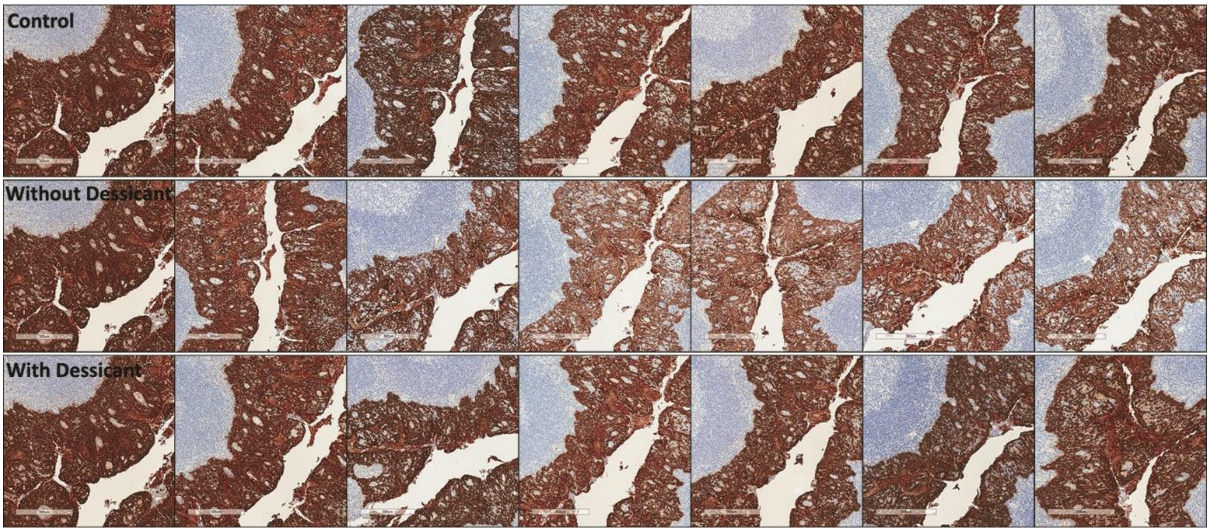

Fig S3 Pan-CK expression (AE1/AE3) in placenta (S3a) and tonsil (S3b) at days 0, 1, 3, 7, 14, 21, and 28. The first row shows tissue sections stored under normal atmospheric conditions, the second and third row show tissue sections within the acceleration chamber at 100% oxygen, 37°C, and 80% humidity without (second row) and with (third row) desiccant.

Supplementary figure S4

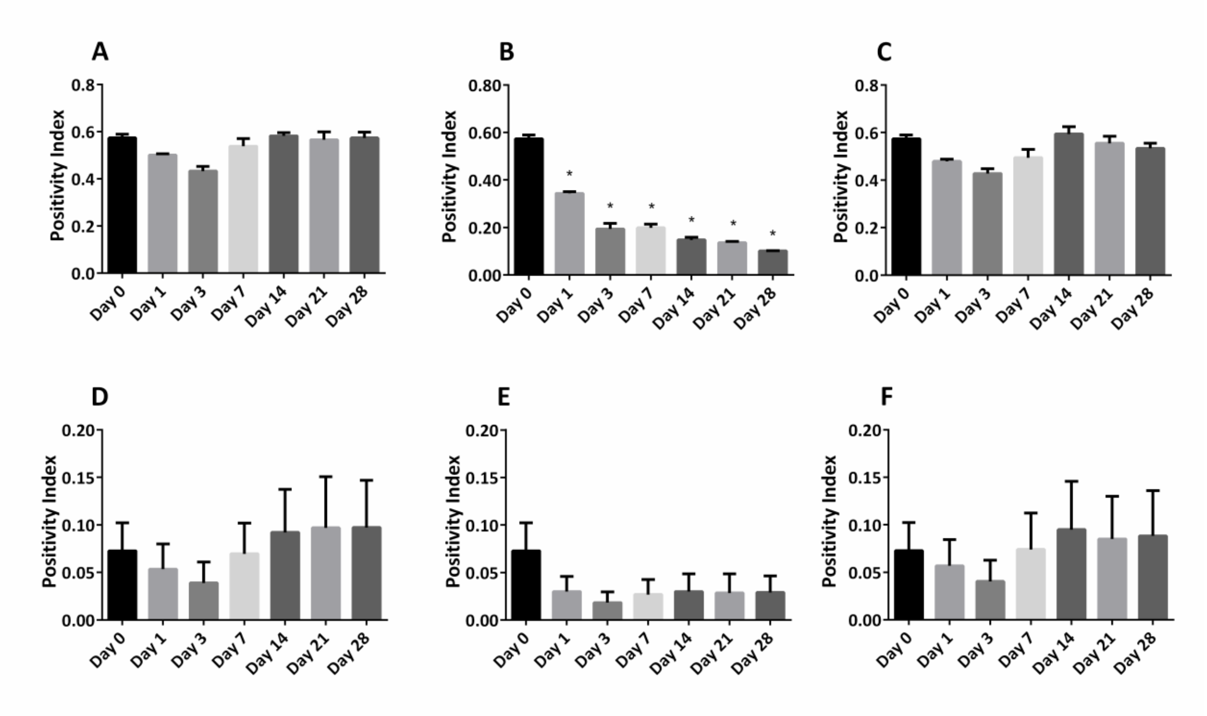

Fig S4. PD-L1 expression by positive pixel count in placenta (A-C) and tonsil (D-F). Control conditions (A + D): 20°C, atmospheric humidity and oxygen. Case sections stored in the acceleration chamber with conditions of 100% oxygen, 80% humidity, and 37°C without (B + E) or with (C + F) desiccant. B and E represent sections stored without desiccant and demonstrate a noticeable loss of PD-L1 signal, significant for placenta and non-statistically significant for tonsil (likely due to small sample size). Similar patterns are seen between the control sections and those stored with desiccant for both placenta and tonsil. Significant reduction in PD-L1 expression is measured against day 0 values. Bars represent mean ± SEM. *p<0.05.
PD-L1, programmed-death ligand-1.

Supplementary figure S5

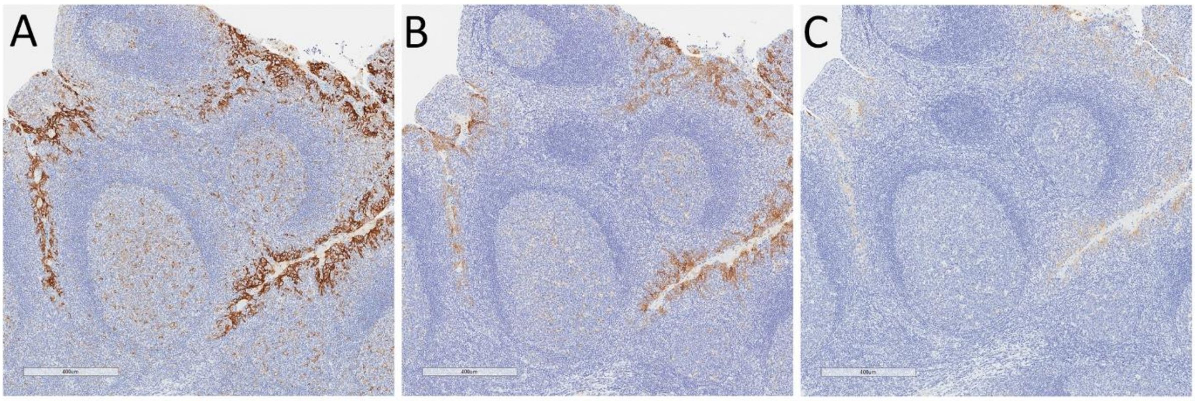

Fig S5. Tonsil tissue stained for PD-L1 with E1L3N in sections stored in the acceleration chamber with conditions of 100% oxygen, 80% humidity and 37°C at day 0 (A), day 14 (B), and day 28 (C). Loss of both crypt epithelial and germinal center staining is seen.
PD-L1, programmed-death ligand-1.

Supplementary figure S6

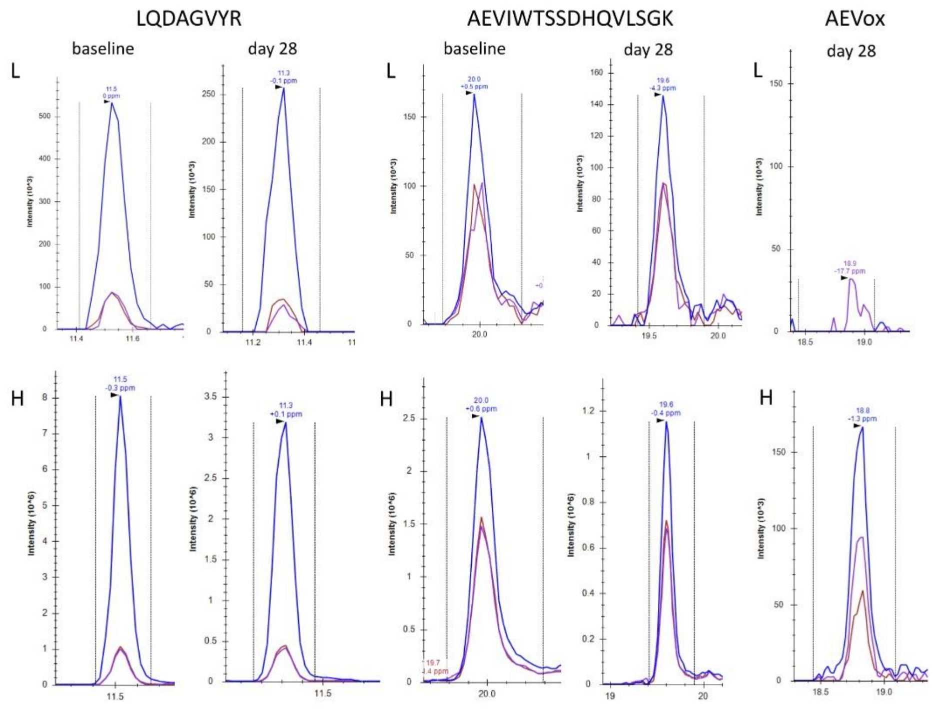

Figure S6. Targeted MS analyses of PD-L1 peptides LQDAGVYR and AEVIWTSSDHQVLSGK and the kynurenine oxidation product AEVI[W+4]TSSDHQVLSGK (AEVox). Each panel depicts the MS signal traces for three most abundant fragment ions for the peptide sequences. For LQDAGVYR, the singly charged y6, y5, and y4 ions from the doubly charged precursor were monitored; for AEVIWTSSDHQVLSGK and AEVI[W+4]TSSDHQVLSGK, the doubly charged y14, y13, and y12 ions from the triply charged precursor were monitored. The upper traces are from the unlabelled peptides derived from endogenous PD-L1. The lower traces are from the isotope labelled standards. Traces for LQDAGVYR and AEVIWTSSDHQVLSGK are shown for representative baseline and day 28 of accelerated degradation. For all peptides, a confirmed detection required co-elution of all three monitored fragment ions with signals from the isotope labelled standards. No significant signal was detected for endogenous AEVox peptide at day 28.
PD-L1, programmed-death ligand-1.

Supplementary figure S7

01

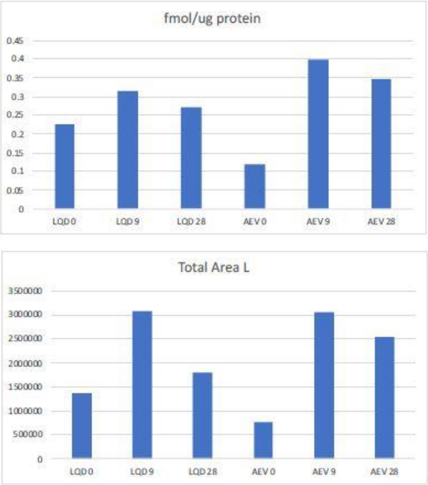

02

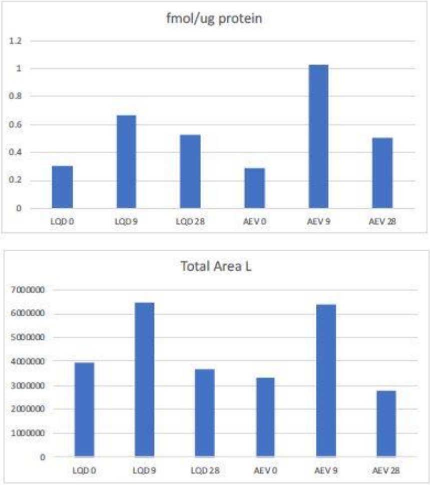

03

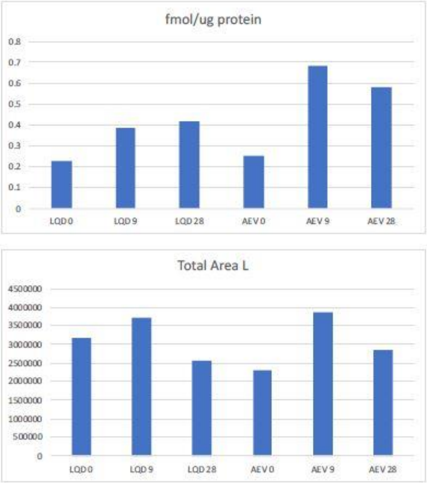

04

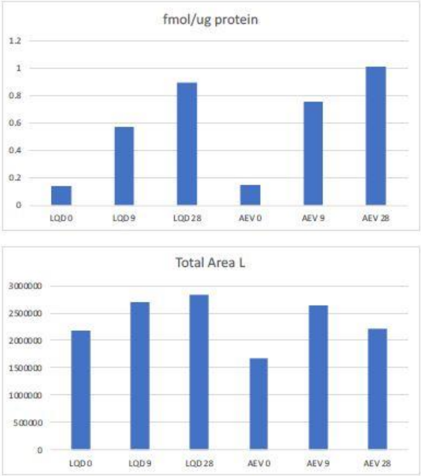

05

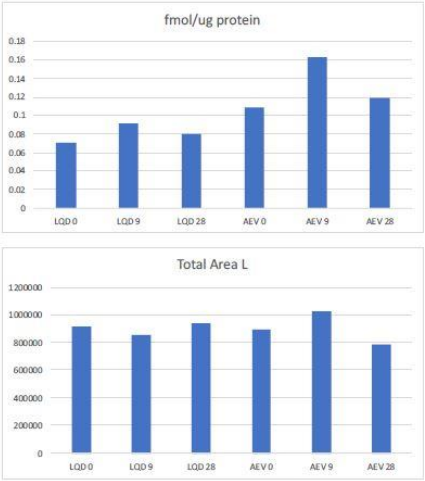

06

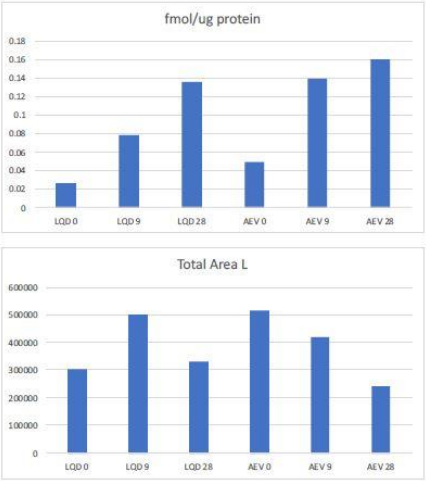

07

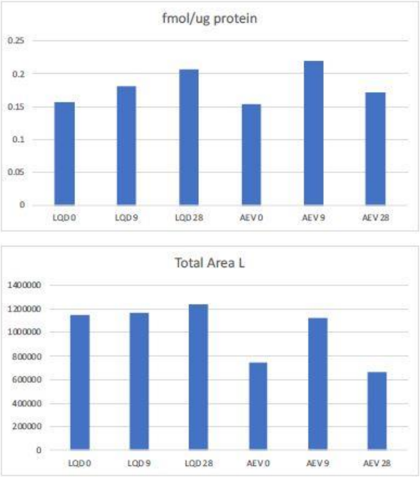

08

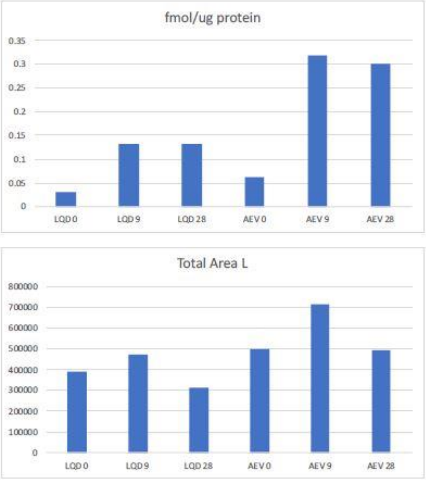

09

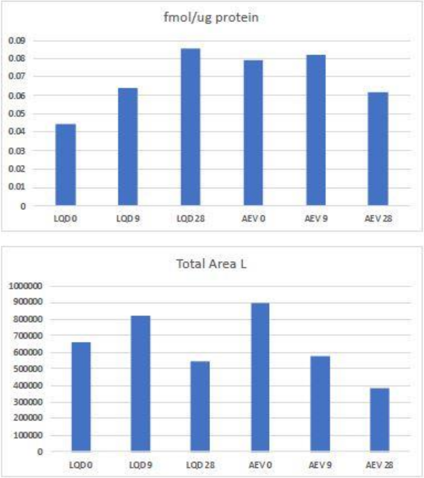

10

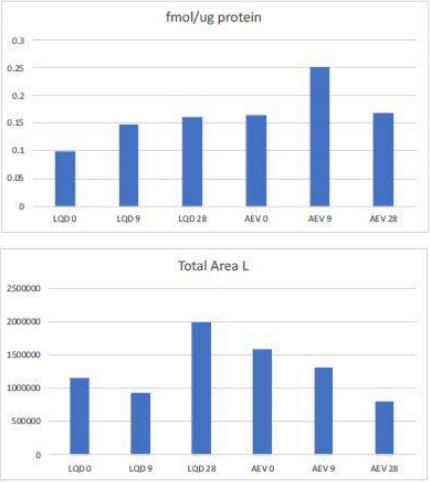

Figure S7. Comparison of calculated PD-L1 amounts (upper) to MS peak areas for unlabeled (endogenous) PD-L1 peptides in 10 FFPE NSCLC samples from accelerated oxidation experiment. PD-L1 amounts were calculated from ratio of the MS peak areas for unlabeled (endogenous) PD-L1 peptides to MS peak areas for the stable isotope labeled internal standards.
PD-L1, programmed-death ligand-1; MS, mass-spectrometry; FFPE, formalin fixed paraffin embedded; NSCLC, non-small cell lung cancer.

Supplementary figure S8

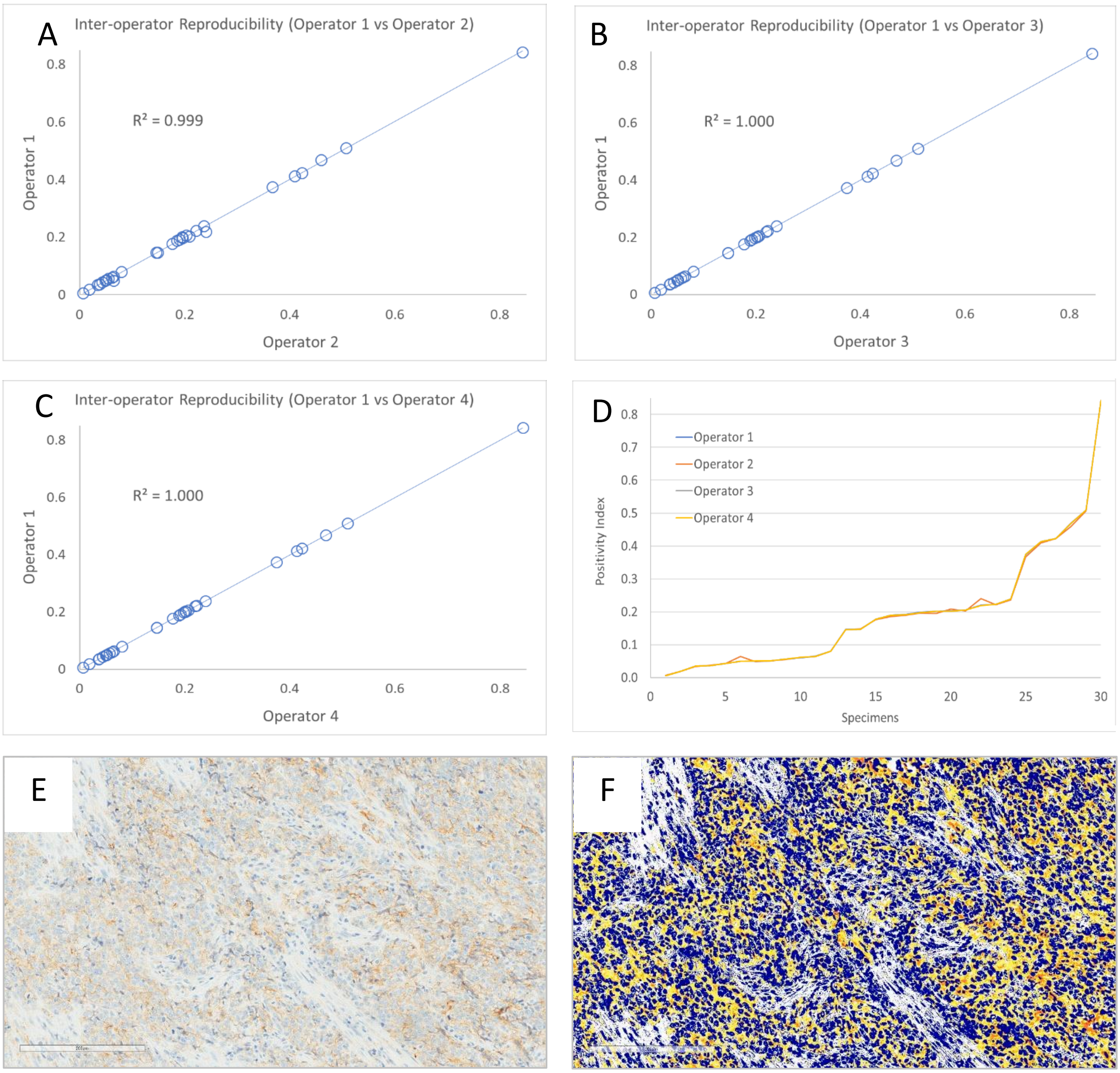

Fig S8. Inter-operator reproducibility of the Positive Pixel Count algorithm. Operators were instructed to analyze the total tumor tissue area from the baseline gastric cancer TMA specimens and assessed with E1L3N as illustrated in Figure 1 A-C (n=30) using the 'Positive Pixel Count v9'[31] algorithm. Scores are given as absolute values (positivity index). Individual inter-operator comparisons are displayed (A, B, and C) and specimens were rank ordered by the positivity score produced by Operator 1 as a benchmark to generate the plot shown in D. Representative images of stained tissue and algorithm mark-up from the inter-operator reproducibility study are also shown (E and F).
TMA, tissue microarray.

Supplementary table S1

Specimens	Positivity Score (Operator 1)	Positivity Score (Operator 2)	Positivity Score (Operator 3)	Positivity Score (Operator 4)
1	0.006146795	0.006394991	0.006182171	0.006142242
2	0.018441975	0.018495963	0.018310262	0.018441975
3	0.035361264	0.034147631	0.035359317	0.035361278
4	0.036747002	0.037464862	0.036509429	0.036697138
5	0.043257868	0.042483261	0.043258597	0.043257636
6	0.049698179	0.064425208	0.049702679	0.049704517
7	0.049862213	0.048903981	0.049517761	0.050458489
8	0.051351711	0.050411937	0.051351101	0.051349771
9	0.056750365	0.054872527	0.056754132	0.056751691
10	0.060755404	0.061486579	0.060787128	0.061203192
11	0.064326789	0.064163024	0.065147008	0.064332527
12	0.080071524	0.079695027	0.080060127	0.080062055
13	0.146111966	0.145285477	0.146314269	0.146116080
14	0.146274755	0.148148996	0.146366286	0.146275163
15	0.177310726	0.176032182	0.177421857	0.177294644
16	0.189053090	0.185312484	0.189053326	0.189054035
17	0.192635355	0.190001075	0.191730750	0.191512173
18	0.198205034	0.196039344	0.198124289	0.198124078
19	0.201710961	0.194405022	0.201704071	0.201109742
20	0.202517892	0.208807548	0.203414053	0.201696845
21	0.206113390	0.202535644	0.206134040	0.206115075
22	0.219934662	0.239959908	0.220200505	0.219276826
23	0.222617117	0.221779645	0.222618116	0.222618886
24	0.239164129	0.236792284	0.239147216	0.239145208
25	0.373808376	0.366824694	0.374208147	0.374097189
26	0.413343702	0.408778088	0.413295209	0.413332678
27	0.423032942	0.422993555	0.423035284	0.423034953
28	0.468177830	0.459173888	0.468839267	0.468166829
29	0.509616837	0.506718780	0.509616874	0.509617093
30	0.843064783	0.842999208	0.843064333	0.843064675

Table S1. Absolute values (positivity index) from Inter-operator Reproducibility Study.

Supplementary figure S9

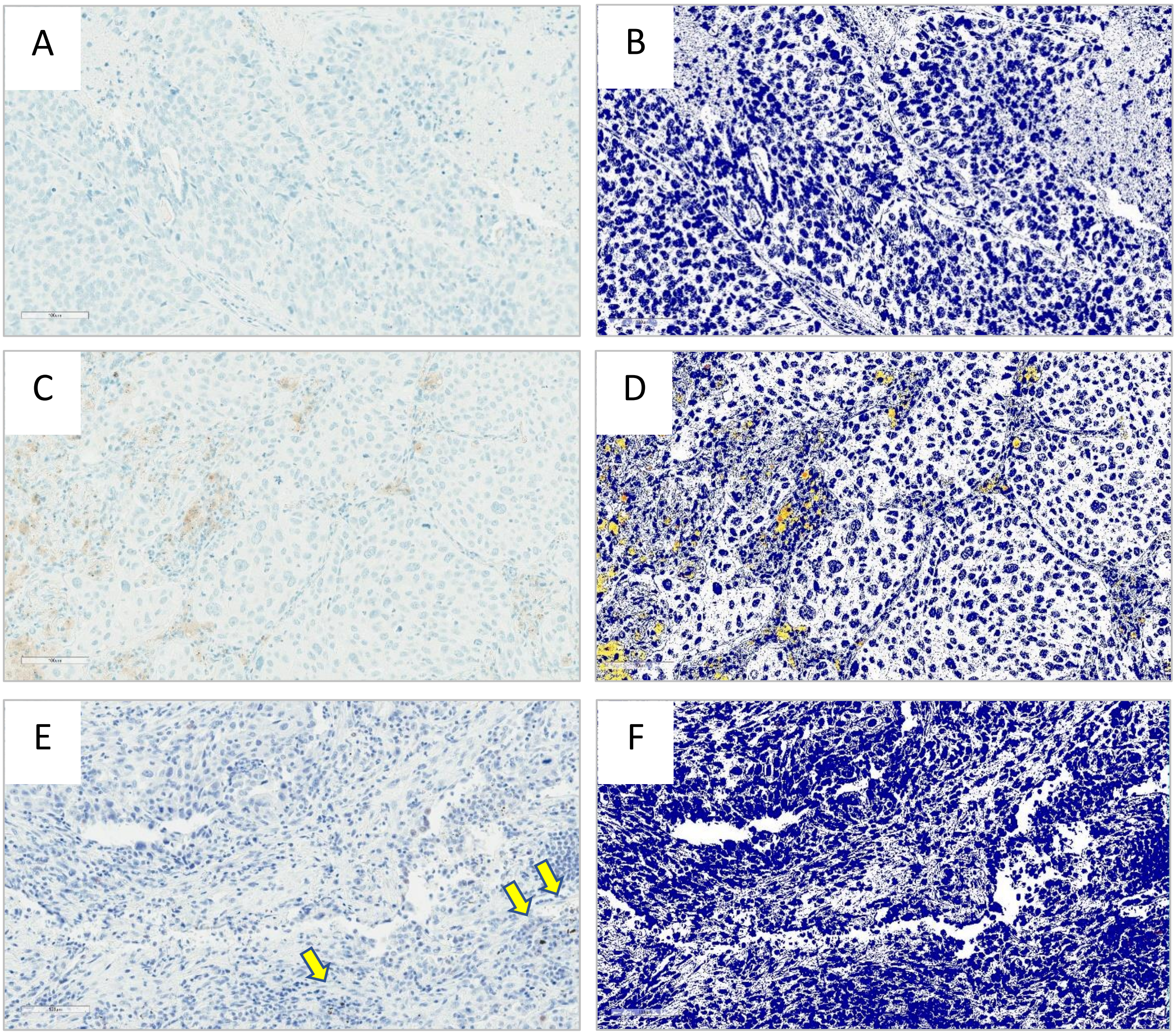

Fig S9. Representative negative control examples assessed with the ‘Positive Pixel Count v9’[31] algorithm. A typical negative control image (A) with algorithm mark-up (B). Examples of negative controls with tissue intrinsic pigmentation due to entrapped pulmonary alveolar macrophages (C) and anthracosis (yellow arrows, E) are detected to a limited extent as shown on their respective mark-up image (D and F). While the positivity score produced from a tissue section can be affected by the presence of tissue intrinsic pigmentation, studies designed to compare the effect of signal loss over a time course and between conditions would be minimally impacted as comparisons across classes were assessed from sections cut from the same tissue specimens.

Supplementary table S2.

Negative Control Examples	Positivity Score	Average
1	0.008383923	0.015146010
2	0.056559483	**Standard Error of the Mean**
3	0.003477672	0.003775963
4	0.003187431	
5	0.006482981	
6	0.003669668	
7	0.000480219	
8	0.007209627	
9	0.040498101	
10	0.021510993	

Table S2. Absolute values (positivity index) from Example Negative Control Specimens.
